# Effect of Ultraviolet Irradiation on 4H-SiC PiN Diodes Characteristics

**DOI:** 10.1186/s11671-021-03601-3

**Published:** 2021-09-10

**Authors:** Xingliang Xu, Lin Zhang, Peng Dong, Zhiqiang Li, Lianghui Li, Juntao Li, Jian Zhang

**Affiliations:** 1 Microsystem and Terahertz Research Center, China Academy of Engineering Physics, Chengdu, China; 2 Institute of Electronic Engineering, China Academy of Engineering Physics, Mianyang, China; 3 School of Electronic Science and Engineering, University of Electronic Science and Technology of China, Chengdu, China

**Keywords:** Silicon carbide, PiN diode, High voltage, Ultraviolet irradiation

## Abstract

In this paper, the effect of ultraviolet (UV) irradiation on the static characteristics of high voltage 4H-SiC PiN is investigated. No significant change is observed in the forward on state characteristic of 4H-SiC PiN diodes before and after ultraviolet light irradiation. However, it is found that the blocking voltage is significantly increased with UV irradiation, which is resulted from the depletion region width extension with the collection of positive charges under the increase of the surface negative charge density. The deep level transient spectroscopy reveals that the UV irradiation induced deep-level defects play a dominant role over the trapped negative charges, and therefore leads to the increase of blocking voltage of 4H-SiC PiN Diodes.

## Introduction

Silicon Carbide (SiC) is expected to be a promising candidate material for the next-generation high-power and high-temperature electronic devices, due to its wide bandgap, high critical electric field strength, high electron saturation velocity and superior thermal conductivity [1–4]. SiC devices are being developed to replace many of the devices currently used in silicon, especially in the requirements which need to operate at high voltages and current levels, and at temperatures above 200 °C. Compared with unipolar devices, SiC bipolar devices are attracting considerable interests owing to ultra-high voltage applications because of the conductivity modulation effect in recent years. As a typical bipolar device, high voltage 4H-SiC PiN diode has been demonstrated, which is a potential choice for high voltage rectifier applications, including advanced smart grid, energy storage and pulsed power [5–8]. In the fabrication process of SiC power devices, several plasma processes such as dry etching and sputter deposition are widely used. Several works have been reported on process-induced damage in SiC devices to bring about electrical degradation [9, 10]. In addition, previous studies reveal that UV irradiation significantly reduces the performance of SiC metal–oxide–semiconductor field-effect transistors (MOSFETs) by high-energy ion bombardment and plasma photoemission [11, 12]. Recently, it is reported that pulsed UV laser irradiation on 4H‑SiC metal–oxide–semiconductor (MOS) can induce the near-interface oxide trap and cause device performance drift and reliability degradation [13]. However, to the best of our knowledge, the UV irradiation on SiC PiN devices have not been investigated so far and it is necessary to understand the effects on the SiC PiN device characteristics.

In this study, we investigated the effect of UV irradiation on 4H-SiC PiN diodes the forward and reverse blocking characteristics using 184.9 nm wavelength UV irradiation. The influence of surface charge accumulation on the breakdown voltage of SiC PiN diodes is presented using Technology Computer-Aided Design (TCAD) simulation. SiO_2_/SiC state densities before and after irradiation were characterized by deep-level transient spectroscopy (DLTS) system on SiC MOS. DLTS is widely used to study interface states density (Dit) distribution and deep defects in MOS capacitor [14, 15].

## Methods

**Fig.1:**
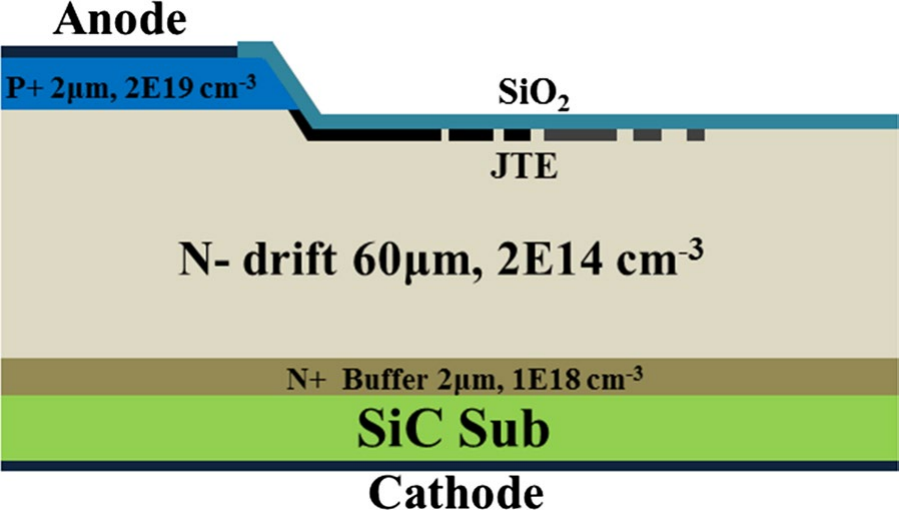
Schematic cross section of 4H-SiC PiN diodes

The schematic structure of the 4H-SiC PiN diode reported in this paper is shown in Fig. 1. The 2-μm-thick buffer layer doped to 1 × 10^18^ cm^−3^ and 60 μm thick n- drift layer with a doping concentration of 2 × 10^14^ cm^−3^ were continuously grown on 4°off-axis heavily doped n-type 4H-SiC(0001) substrates. Then, the top layer was p + anode with 2 μm thick and a doping concentration of 2 × 10^19^ cm^−3^. After epitaxial growth, a circular isolation mesa structure with 2.5 μm height and 300 μm diameter was patterned using inductively coupled plasma reacting through the p + anode layer into the n- drift layer. The etching gases and mask material were SF_6_/O_2_ and deposited by plasma-enhanced chemical vapor deposition, respectively. Following the mesa isolation, a double Al-implant of 1 × 10^17^ cm^−3^ based junction termination extension (JTE) was formed to alleviate electric field crowding near the mesa edge. The implants were activated by annealing in Ar at 1650 °C for 30 min. A sacrificial SiO_2_ layer was grown at 1100 °C for 1 h and dipped with HF to provide a fresh surface for thermal oxidation. Then, thermal oxidation in dry O_2_ ambient was performed at 1100 °C for 3 h with SiO_2_ layers thickness of about 40 nm, followed by annealing in Ar ambient at 1100 °C for 1 h. Contact materials were Ni/Ti/Al for the anode and Ni for the cathode. These metals were annealing at 800 °C and 1000 °C for 2 min to obtain high quality ohmic contact, respectively. The specific contact resistances characterized by linear transfer length method were 1 × 10^−5^ Ω cm^2^ and 3.75 × 10^−5^ Ω cm^2^ for Ni n type and Ni/Ti/Al p type ohmic contact, respectively. Overlayer metal with thick Al was deposited onto the anode and cathode. The silicon dioxide layer and a thick polyimide layer were patterned on the front to avoid surface sparking during high voltage measurements. In addition, the SiC-MOS capacitors were fabricated on a high quality n-type (7 × 10^15^ cm^−3^) epitaxy layer on a heavily doped 4H-SiC substrate. The 40 nm thermal oxide was grown based on standard process of SiC PiN. The gate electrode and backside ohmic contact were formed with Al and Ni, respectively.

4H-SiC PiN diodes and SiC-MOS capacitors were irradiated by UV light using mercury lamp with wavelength of 184.9 nm in air for 72 h without bias stress. The electrical characteristics of 4H-SiC PiN before and after UV irradiation were evaluated by the Wentworth probe and the Agilent B1505A semiconductor characterization system. SiC-MOS capacitors interface states and fixed charges were then characterized by a PhysTech Fourier Transform DLTS system. The applied reverse bias *V*_R_ and the filling pulse voltage *V*_P_ were 15 V and 2 V, respectively. The sampling period *t*_w_ of the bias pulse from *V*_R_ to *V*_P_ was 1.5 s.

## Results and Discussion

**Fig. 2:**
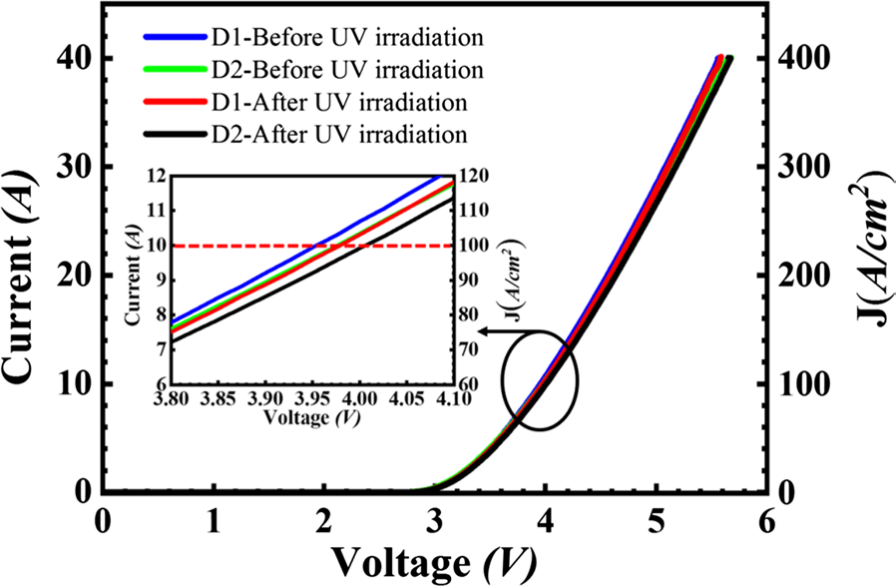
Forward on state characteristic of 4H-SiC PiN diode before and after UV irradiation

**Fig. 3:**
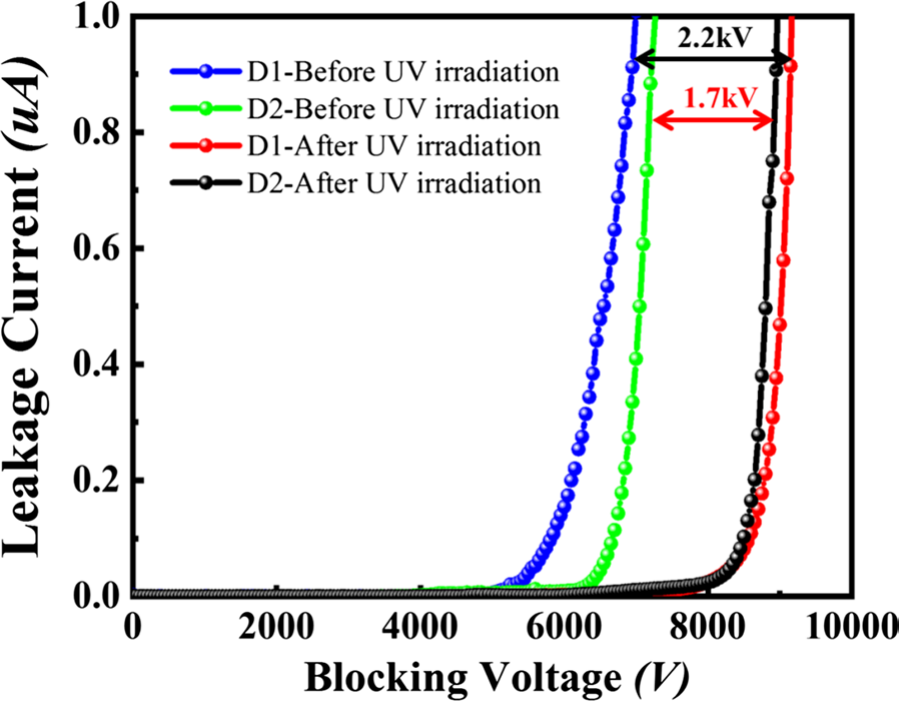
Reverse characteristic of 4H-SiC PiN diode before and after UV irradiation

The effect of UV irradiation on static characteristics of SiC PiN is shown in Figs. 2 and  3 where the characteristics measured before and after irradiation are compared for device 1 (D1) and device 2 (D2). The diameter of circular SiC PiN diodes is 3.5 mm and active area is about 10 mm^2^. The forward voltage drop for SiC PiN is about 3.95 V at current density of 100 A/cm^2^ for both two devices before UV irradiation. It is clearly observed in Fig. 2 that there are no significant changes in the forward on state characteristic for two devices, changing from 3.95 to 4.0 V after UV irradiation. The achieved blocking voltages of device 1 and device 2 fabricated on 60 μm thick n-drift epilayer are 7 kV and 7.2 kV at leakage current of less than 1μA, respectively. It should be noted that the blocking efficiency of PiN diodes is about 70% of theoretical value of 9.7 kV for 60 μm thick drift layer, which the inaccuracy of the impurity activation probably leads to deviation from optimal JTE implant window. After UV irradiation, the blocking voltage of device 1 shows a remarkable increase from 7 to 9.2 kV with 2.2 kV improvement, approaching to the ideal parallel plane value. Correspondingly, 1.7 kV improvement is achieved for device 2 after UV irradiation.

**Fig. 4:**
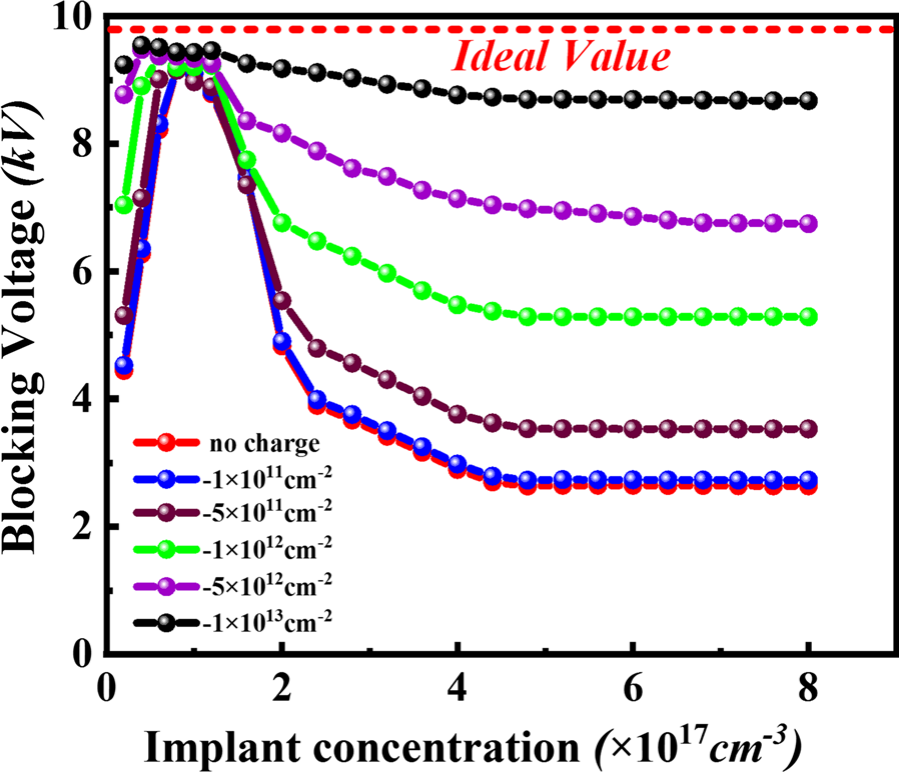
Simulated breakdown voltage versus JTE implant concentration for surface negative charge, including no charge, 1 × 10^11^ cm^−2^, 5 × 10^11^ cm^−2^, 1 × 10^12^ cm^−2^, 5 × 10^12^ cm^−2^, and 1 × 10^13^ cm^−2^ respectively

It is well known that the surface traps have a significant effect on electric field distribution in terminal region and then affect the reverse blocking characteristics in the SiC power device. Ikeguchi et al. have indicated that high-energy UV illumination with photon energy ≥ 5 eV can transform the pre-existing strained C–C double bonds into active electron traps, and thus leads to the production of negatively charged interface defects observed by positive *V*_FB_ shift of C/V curves [11, 12]. Meanwhile, the generated electron by UV irradiation may be captured by deep-trap interface defects and thus get negatively charged with the increase of fixed charge density. Figure 4 illustrates the dependence of simulated blocking characteristics on the implant concentration for double implant JTE SiC PiN diode with various surface negative charges. It is obviously seen that the surface charges in JTE region have a significant effect on the reverse blocking performance, especially when the JTE implant concentration is deviated from optimal window. For a given double implant termination structure, as the increase of surface negative charges, a wider optimum window of target blocking value could be achieved. It is clear that the JTE structure is less sensitive to the interface charge below 1 × 10^11^ cm^−2^ and the blocking voltage has no obvious change. With surface charges density higher than 5 × 10^11^ cm^−2^, the breakdown voltage would dramatically increase. Surprisingly, the breakdown voltage is approximately achieving theoretical value with implant window from 2 × 10^16^ cm^−3^ to 8 × 10^17^ cm^−3^ at surface charges density of 1 × 10^13^ cm^−2^.

**Fig. 5:**
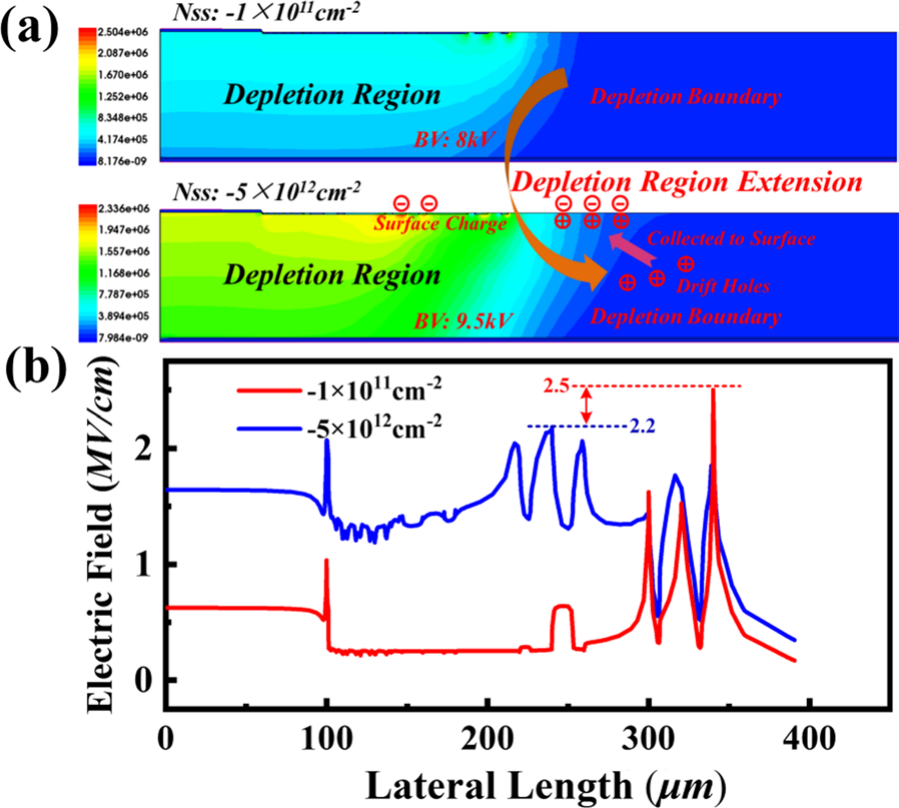
Simulated electric field distributions of SiC PiN with surface negative charge density of 1 × 10^11^ cm^−2^ and 5 × 10^12^ cm^−2^: **a** electric field distribution with the depletion region evolution **b** electric field cutline below the JTE/n-drift region junction. The implant concentration is used 6 × 10^16^ cm^−3^

Figure 5 compares the influence of negative surface charge accumulation on electric-field profile at blocking characteristic. The electric field distribution with the depletion region evolution in the 4H-SiC PiN diodes is shown in Fig. 5a. When the negative charge increases to 5 × 10^12^ cm^−2^ at the SiO_2_/SiC (JTE structure region) interface of SiC PiN, the positive charge of N drift layer is collected to the interface surface, leading to the significant extension of depletion region [16]. The Fig. 5b shows the electric field cutline below the JTE/n-drift region junction with the surface negative charge density of 1 × 10^11^ and 5 × 10^12^ cm^−2^. In the case of low surface charge of 1 × 10^11^ cm^−2^, severe electric field crowding appears in edge termination region with a maximum value of 2.5 MV/cm and breakdown voltage is about 8 kV. As the charges density increases to 5 × 10^12^ cm^−2^, the peak electric field drops to 2.2 MV/cm and the electric field crowding at edge termination region is suppressed in comparison. Meanwhile, the electric field distribution is more uniform, and the breakdown voltage increases evidently. Therefore, surface negative charge can cause the extension of depletion and alleviate electric field crowding, resulting in the improvement of breakdown voltage.

**Fig.6:**
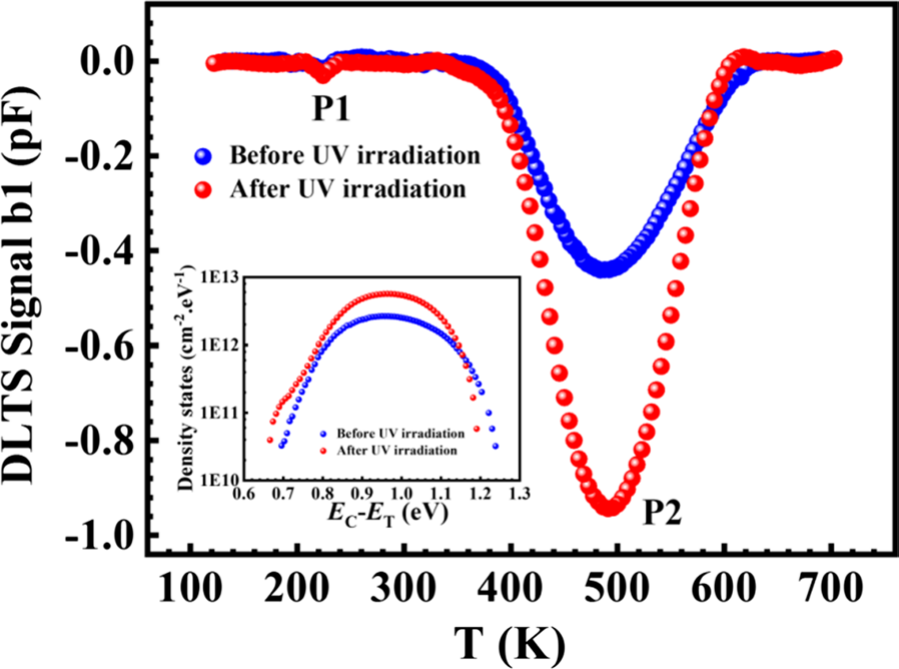
DLTS spectra for 4H-SiC PiN diodes before and after UV irradiation. The insert shows the *D*it distributions for 4H-SiC PiN diodes before and after UV irradiation

To further validate the physical mechanisms of generation of the irradiation induced electronic defects, a better electrical characterization of SiO_2_/SiC interface is required to investigate in more details. DLTS spectra for 4H-SiC SiC-MOS capacitors were characterized in depletion from 15 to 2 V before and after UV irradiation, as shown in Fig. 6. From the DLTS spectra, two peaks were observed in the 4H-SiC MOS capacitors both before and after UV irradiation, locating at 210 K and 490 K, respectively. Negative DLTS peaks indicate that the P1 and P2 level are electron traps. The broad and significant P2 level shows a significantly increase of the peak amplitude, meaning that the concentration of electron trap is increased by UV irradiation. Besides, it is found that the DLTS signal increases proportionally to filling time, exhibiting trap-filling kinetics characteristic of extended defects, such as interface defects rather than point defects. The insert shows the distributions of interface state density versus activation energy E_T_. The interface state density is calculated by $D_{{{\text{it}}}} = \varepsilon_{{{\text{sic}}}} C_{{{\text{ACC}}}} AN_{{\text{D}}} \Delta C/\left[ {C_{R}^{3} kT} \right]$ [17]. It can be seen from the figure that the interface defect gives rise to an energy band in the bandgap from *E*_C_ − 0.65 eV to *E*_C_ − 1.25 eV and its density significantly increased from 2 × 10^12^ cm^−2^ eV^−1^ to 6 × 10^12^ cm^−2^ eV^−1^ after UV irradiation. Combining transient capacitance measurements, high-resolution transmission electron microscopy and density-functional-theory calculations, Dong et al. suggested that this interface defects originated from negatively charged excess split-interstitial carbon at the interface [18]. The P1 peak at 210 k corresponds to an electron trap at *E*_C_ − 0.41 eV. Its concentration exhibits no essential change after UV irradiation, and tentatively assigned P1 trap to point defects in the SiC epilayer. However, its atomic configuration is still unclear and needs to be clarified in future investigation.

## Conclusions

The influence of UV irradiation on the electrical characteristics of 4H-SiC PiN diodes has been investigated. From the results of electrical experiments, insignificant change of the forward current is observed after UV irradiation. However, the UV irradiation appears a remarkable increase of the blocking voltage. It is found the UV irradiation generates deep level defects in the interface of PiN diodes, resulting in increase of the deep acceptor levels in the bandgap. These deep-level defects serve as the center of electron capturing and lead to the significant increase of negative charge in SiO_2_/SiC interface. The positive charges of N dirft layer are collected to the interface surface, and further promote the extension of depletion region with more uniform electrical field distribution, which bring about the increase of blocking voltage.

## Data Availability

All the data are available without restrictions.
